# Predicting the impact of household contact and mass chemoprophylaxis on future new leprosy cases in South Tarawa, Kiribati: A modelling study

**DOI:** 10.1371/journal.pntd.0007646

**Published:** 2019-09-20

**Authors:** Charlotte Gilkison, Stephen Chambers, David J. Blok, Jan Hendrik Richardus, Eretii Timeon, Erei Rimon, Patricia Priest

**Affiliations:** 1 Department of Preventive and Social Medicine, University of Otago, Dunedin, New Zealand; 2 Department of Pathology, University of Otago, Christchurch, New Zealand; 3 Department of Public Health, Erasmus MC, University Medical Center Rotterdam, Rotterdam, The Netherlands; 4 Department of Public Health, Ministry of Health and Medical Services, Bikenibeu, Kiribati; 5 Department of Nursing, Ministry of Health and Medical Services, Bikenibeu, Kiribati; Hospital Infantil de Mexico Federico Gomez, UNITED STATES

## Abstract

**Background:**

The country of Kiribati is a small Pacific island nation which had a new case detection rate of 191 per 100,000 in 2016, and is one of the few countries yet to reach the WHO leprosy elimination goal. Chemoprophylaxis of household contacts of new cases, or to the whole population in a highly endemic areas have been found to be effective in reducing new case rates. This study investigated the potential impact of different chemoprophylaxis strategies on future cases in South Tarawa, the main population centre of Kiribati.

**Methodology:**

The microsimulation model SIMCOLEP was calibrated to simulate the South Tarawa population and past leprosy control activities, and replicate annual new cases from 1989 to 2016. The impact of six different strategies for delivering one round of single dose rifampicin (SDR) chemoprophylaxis to household contacts of new cases and/or one or three rounds of SDR to the whole population was modelled from 2017 to 2030.

**Principal Findings:**

Our model predicted that continuing the existing control program of high levels of public awareness, passive case detection, and treatment with multidrug treatment would lead to a substantial reduction in cases but this was less effective than all modelled intervention scenarios. Mass chemoprophylaxis led to a faster initial decline in cases than household contact chemoprophylaxis alone, however the decline under the latter was sustained for longer. The greatest cumulative impact was for household contact chemoprophylaxis with three rounds of mass chemoprophylaxis at one-year intervals.

**Conclusions:**

The results suggest that control of leprosy would be achieved most rapidly with a combination of intensive population-based and household chemoprophylaxis. These findings may be generalisable to other countries where crowding places social contacts as well as household contacts of cases at risk of developing leprosy.

## Author summary

Leprosy rates in Kiribati are some of the highest in the world and it is one of the few countries yet to reach the World Health Organization leprosy elimination goal of a prevalence of less than one case per 10,000 population. The greatest burden is in the capital South Tarawa and the connected islet of Betio. Interest has increased for the use of chemoprophylaxis, the administration of preventive antibiotics to apparently healthy individuals who may be incubating the disease, which has been demonstrated to be effective in both household contacts and the whole population in two recent studies. In this study we used the individual-based model SIMCOLEP to predict the impact of six difference scenarios using single dose rifampicin (SDR) chemoprophylaxis in household contacts and/or the entire population on future new cases in South Tarawa. We found that all chemoprophylaxis strategies were predicted to be more effective than the current control strategy, particularly a combination of household contact chemoprophylaxis alongside three rounds given to the entire population in consecutive years.

## Introduction

Despite being one of the most ancient diseases, there are annually still around 200,000 newly diagnosed leprosy cases worldwide [1]. Leprosy is one of the neglected tropical diseases, affecting the poorest and least-developed countries [2]. Most cases are in India, Brazil, and Indonesia, however 22 countries are classified as “high burden” by the World Health Organization (WHO) [3].

Kiribati, formerly known as the Gilbert Islands (or Gilbert and Ellice Islands), is a country of 33 coral atolls and islands spanning an area of 3.5 million square kilometres of the Pacific Ocean. The widely dispersed population of 110,136 (2015) [4] provides challenges to delivering effective health care because of high transport and shipping costs, and limited communication infrastructure.

Kiribati reached the WHO leprosy elimination goal of less than one case per 10,000 population in 2000, however this was unable to be sustained. The new case detection rate (NCDR) in 2016 was 191 per 100,000 [1]. There is a relatively high proportion of paucibacillary (PB) disease, a low rate of grade-2-disability, and ongoing spread including to children [3]. The high NCDR has led the Ministry of Health and Medical Services (MHMS) in Kiribati to identify control of leprosy as a priority in their national development plan [5]. To reduce the high rates, the Kiribati MHMS has partnered with the Pacific Leprosy Foundation (PLF) to improve several aspects of the programme, including resourcing, database management, diagnostic skills, intensive case finding, improved publicity campaigns, and follow-up of patients and contact tracing. This has led to an increase in reported cases in recent years.

South Tarawa is the political and economic centre of the country. Along with Betio, an islet connected to South Tarawa by a causeway, South Tarawa has experienced major population expansion through high birth rates and internal migration. Currently about half the population lives there, but the very limited land for settlement has caused increasing crowding in the heavily populated areas, with population densities in 2015 of 2,772 people per km^2^ in South Tarawa, and 10,377 people per km^2^ in Betio [4]. Land constraints have been exacerbated by the threat of sea level rise from climate change.

Chemoprophylaxis aims to prevent the development of symptomatic disease in those with subclinical disease. Early research interest in chemoprophylaxis for leprosy waned with the introduction of multidrug therapy (MDT), but has recently increased again. In 2005 Bakker et al. found a 75% reduction in new cases when two doses of rifampicin was given to the entire population of an isolated highly leprosy-endemic Indonesian island, while chemoprophylaxis of household contacts was not effective in this setting [6]. In 2008 the COLEP trials investigated the impact of single dose rifampicin (SDR) in spatially and genetically defined contacts [7]. They found an average reduction of 57% after two years. Both studies found that chemoprophylaxis was most effective in more distant contacts, likely because their subclinical infection is at an earlier stage, having had less intense exposure.

Despite a greater risk of acquiring leprosy in household contacts, research has found that up to 75% of new cases in a high prevalence area had no known index case [8]. Recent recommendations have been for leprosy control to be focused on high risk contacts as leprosy has become rarer, with the exception of some smaller areas of high prevalence [9]. This has significant implications on whether a focused household or mass chemoprophylaxis would be beneficial in the population.

SIMCOLEP is a leprosy microsimulation model which simulates individual life and disease histories [10]. The use of a mathematical models allows for the impact of interventions to be predicted in a specific population over a long time frame, based on past and current epidemiological data. This is important for leprosy as the long incubation period means that it can take many years for the effect of interventions to be visible. Previous applications have been the modelling of household chemoprophylaxis in Bangladesh [11] and Brazil [12], and the prediction of country-level elimination in India, Brazil and Indonesia.

The Kiribati MHMS wished to determine whether chemoprophylaxis could contribute to the control and potential elimination of leprosy in Kiribati. We adapted the SIMCOLEP microsimulation to simulate the demographic characteristics and leprosy control program in Kiribati over time, fitting it to the leprosy new case trend from 1989. It was then used to compare the predicted effectiveness of household contact, mass, and combined household and mass chemoprophylaxis strategies in reducing future leprosy cases.

## Methods

### Ethics Statement

The study protocol was reviewed by the senior clinical management team of the MHMS in Kiribati and University of Otago Human Ethics committee where it was regarded as minimal risk health research not requiring informed consent (June 2018).

### SIMCOLEP model

SIMCOLEP is a stochastic individual-based model that simulates a closed population of fictitious individuals structured into households [10]. These individuals each have a life history containing birth, death, marriage, and children. Households form and dissolve during the simulation with the movement of individuals or couples. The life histories modelled reflect, as far as possible, the realities in the country which is being modelled.

The transmission of *Mycobacterium leprae* is modelled through two processes; general population and within-household. Transmission occurs by direct contact with an infectious person, the rate of which is different in the general population and within-households. The contact rate and the probability of infection during contact determines infectivity.

There are two distinct forms of leprosy in this model: PB, which self-heals after some time and multibacillary (MB), the latter being the only infectious form in this model. The natural history of infection is the same as in a previous leprosy model SIMLEP [13]. Susceptibility of individuals was randomly assigned at birth and we assumed that 20% of individuals are susceptible based on the results of model fitting with SIMCOLEP in a previous study [10]. The type of leprosy is randomly determined and in keeping with the Kiribati pattern we assigned two-thirds of cases to be PB.

Adapting SIMCOLEP to the situation in South Tarawa was carried out by using available data on the demography of Kiribati, the epidemiology of leprosy there, the history of leprosy control measures, and the calibration of parameters whose values are unmeasured or unmeasurable. Through calibration we derived optimal values for the household parameters, and a range of values for epidemiological parameters. For our predictions, we used the optimal values for household parameters and sampled uniformly from the derived range of values for the epidemiological parameters. This allows our predictions to account for the uncertainty in epidemiological parameter values.

#### Demography of Kiribati

The model was quantified with demographic data for population growth rate, fertility and survival rates, age and sex distribution, and the fraction of married population per age group. This was using country-specific data from the census and the United Nations (S1 Table). The model was then fitted to the household size distribution in South Tarawa. The parameters that determine household size, such as those relating to the movement of single and married people, were calibrated so that the household size distribution in the model was the same as the urban household size distribution reported by the Kiribati Demographic and Health Survey 2009 [14] using chi-square minimization. Only those parameters that were essential to replicate household distribution were calibrated (S2 Table); the proportion of single males aged 12–22 that moved households, the maximum household size that a single male would move into, and the average number of years before a married couple leaves the parental household to form their own two-person household. Using the Pearson’s Chi-squared Test a p-value > 0.05 was calculated, indicating that the simulated distribution was not significantly different than the observed (S1 Fig).

#### Leprosy control measures

After the model was fitted to the demographics of South Tarawa, the annual number of new cases in the model were fitted to the new cases from 1989 to 2016 recorded in the Kiribati Leprosy Unit database. When the relevant data was available it was used as direct inputs into the model (Table 1).

**Table 1:** Overview of leprosy parameters to quantify the model.

Parameters	Values	Source
**Natural history of infection**		
Susceptible	20%: random mechanism	Fischer et al. 2010 [10]
MB:PB ratio	1:2	Pacific Leprosy Foundation database
PB subclinical duration mean (gamma distributed)	4.2 years, SD: 1.9	Fischer et al.
PB self-healing mean (exponentially distributed)	5 years	Fischer et al.
MB subclinical duration mean (gamma distributed)	11.1 years, SD: 5	Fischer et al.
**Treatment**		Blok et al. 2015 [15]
Dapsone use	1970–1990	
Dapsone relapse rate	0.015 per year	
MDT use	1990 onwards	
MDT relapse rate	0.01 per year	
Relapse	To MB: 90% To PB: 10%	
**Control**		
Mass chemoprophylaxis 1996–1997 coverage	85%	Daulako et al.1999 [16]
Active case detection probability for mass screening 1996–1997	0.13 (0.106–0.154)	Calibrated
BCG protection	60%	Schuring et al.] 2009 [17]
*Passive detection delay (years)*		
1970	50	Assumption
1985	24.3 (21.6–27.0)	Calibrated
2009	14.1 (12.7–15.6)	
2010	5.3 (4.9–5.8)	Calibrated
2016	1.1 (0.9–1.3)	Calibrated
**Transmission**		
*Infectivity function*		Meima et al. 2004 [13]
PB	0	
Asymptomatic MB	Linearly from 0 to 1	
Symptomatic MB	1.0	
*Contact rate*		
General population (C_pop)_	0.59 (0.57–0.61)	Calibrated
Within-household (C_hh)_	0.98	Fischer et al. 2010 [10]

Information on control activities from 2009 onwards was obtained from records provided by the PLF. Dapsone monotherapy began in 1970, and MDT was introduced in 1989. In South Tarawa, infant BCG immunization began in 1980, and in 2014 had a coverage of 72%. The BCG vaccination is assumed to have a 60% protective effect based on a meta-analysis [18]. Daulako et al. conducted two rounds of mass screening in South Tarawa, with mass chemoprophylaxis in the second round in 1997 [16]. Contact tracing was introduced in South Tarawa in 2009 but implementation was inconsistent resulting in poor coverage so this could not be included in the model. The previously calibrated optimal within-household contact rate (C_hh_) of 0.98 was used with the assumption that within-household contact in South Tarawa is the same as in Bangladesh [10].

The goodness-of-fit of calibrated epidemiological parameters was assessed using log-likelihoods assuming a Poisson distribution (Table 1). The general population contact rate (C_pop_) was calibrated to match the endemicity level in terms of new cases detected. The coverage of active case detection for both MB and PB cases during the two rounds of mass screening by Daulako et al. was calibrated to fit the peak in cases in 1997. Changes in the number of new cases identified through voluntary presentation to health services for diagnosis were modelled by varying the assumed passive detection delay (the number of years between development of symptoms and diagnosis through passive detection). In 1985, there were increases in worldwide leprosy efforts, so this was the first calibrated passive detection delay. Reports written by consultant leprologists for the PLF detail efforts to increase awareness from 2009 onwards, and thus 2009 and 2010 passive detection delays were calibrated. The 2016 decrease in passive detection delay was the result of awareness campaigns to encourage participation at skin camps for diagnosis. Further details of the fitting procedure can be found in Fischer et al [10].

### Model fitting

First we fitted the model to the years 1989 to 2000, and omitted the remaining years. Short-term prediction for the years 2001 to 2003 were compared to data to validate the model. The model was deemed valid when it was able to predict the number of cases from 2001 to 2003 with sufficient accuracy to lie within the confidence interval for the prediction. We did not include the years after 2004 because these trend is confounded by substantial changes in the leprosy control situation. Then we fitted the model to all the data, and forward predictions were based on the final fit.

#### Predictions and interventions

Predictions under all scenarios were made to 2030 to determine their impacts about ten years after their introduction. The baseline control program is the current leprosy situation and control programs as at 2016. This included treatment with MDT, infant BCG vaccination, high levels of passive case detection, but no active case detection. SDR is simulated to have a 50% protective effect in all individuals, the average protective effect from the COLEP trial. All intervention scenarios simulated introduced the intervention in 2017 alongside the continuation of the baseline control program. We investigated the impact of ongoing household contact chemoprophylaxis (‘household’) and rounds of mass chemoprophylaxis (‘mass’) strategies on transmission in six distinct scenarios; 1) household only; 2) one round of mass only; 3) three rounds of mass only, in consecutive years; 4) one round of mass with household; 5) three rounds of mass in consecutive years with household; 6) three rounds of mass in alternate years with household. To explore the impact of coverage, all scenarios were simulated at 100% and 80% coverage of individuals. The output of the model is the annual new diagnosed cases.

Simulations were conducted by using optimal values for household parameters and by sampling from the range of calibrated values for leprosy parameters to create 1000 unique parameter sets. Predictions were the average output of these 1000 simulations. We used R software [19] for analysis of simulated data and figure generation.

## Results

**Fig 1:**
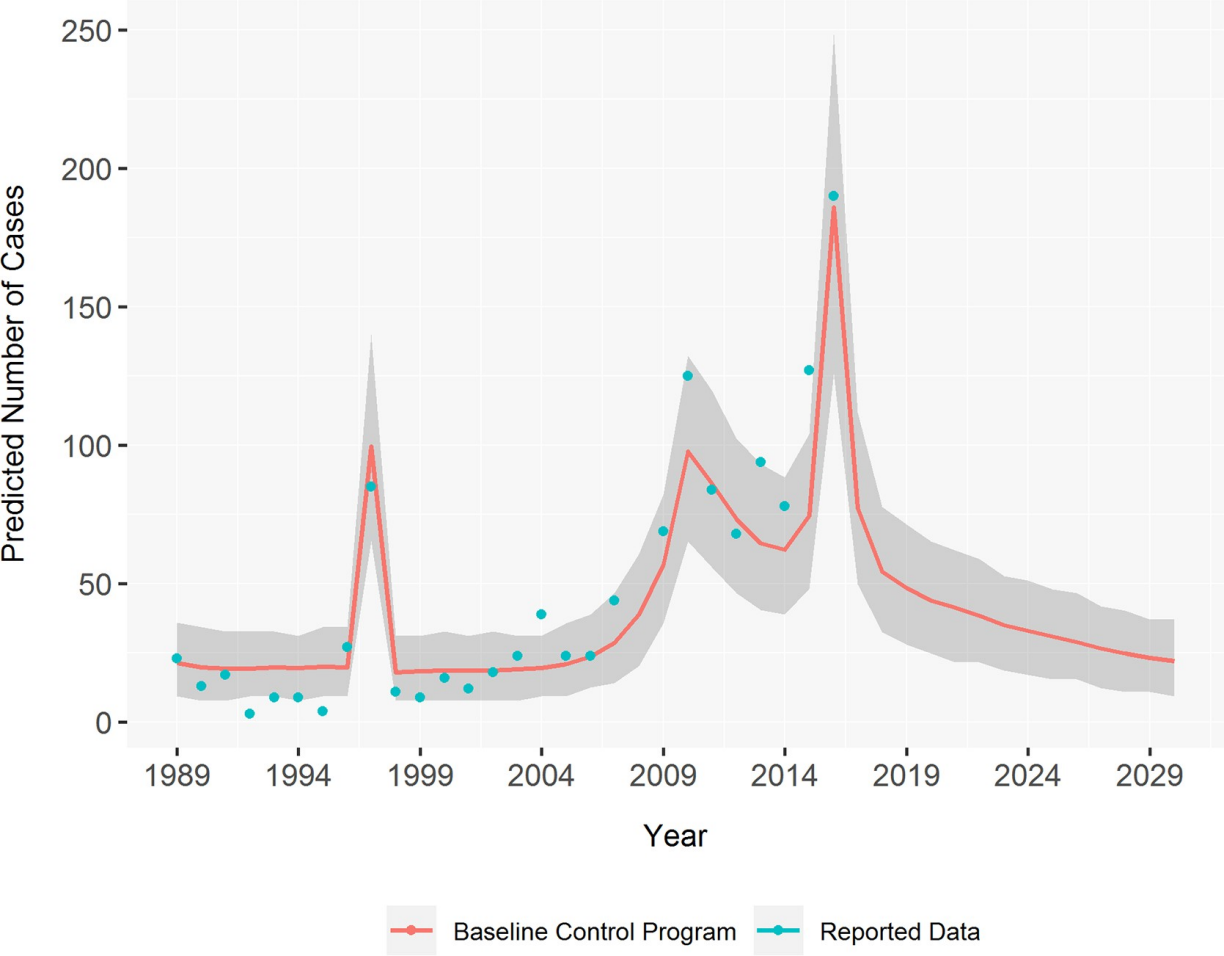
Predicted new leprosy cases in South Tarawa from 1989 to 2030. The model was fitted to the observed cases from 1989 to 2016. Results are the average of 1000 runs. The shaded area is the confidence interval, representing the stochasticity of each run.

A good model fit to the observed leprosy trend was achieved through calibration (Fig 1). The model predicts a substantial decrease in cases by continuing the baseline control program. From 2017 to 2030 the number of new leprosy cases will decrease by 88.2% (95% CI: 85.0–91.4). The greatest drop is from 2016 to 2017, with a predicted decrease of 58.4% (95% CI: 55.0–60.5).

**Fig 2:**
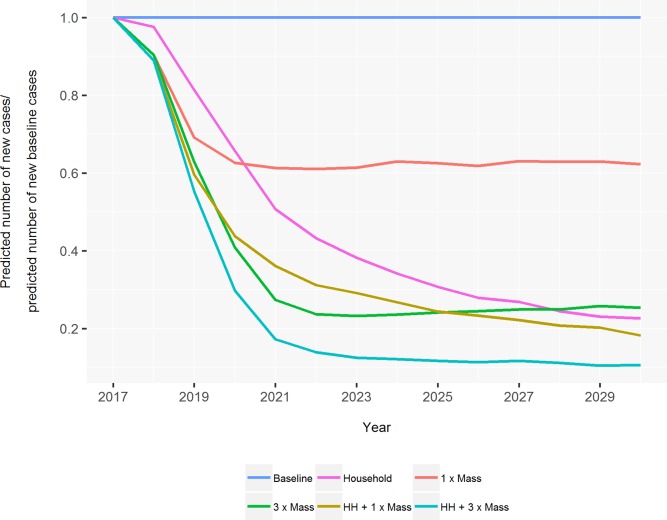
The predicted impact of six chemoprophylaxis scenarios on future cases in South Tarawa from 2017 to 2030. This figure shows the future number of cases for each chemoprophylaxis scenario relative to predictions under the baseline control program only. Results are an average of 1000 runs of the model.

All intervention scenarios are predicted to lead to an even greater reduction from first introduction and over the entire prediction period (Fig 2), demonstrating the benefit of chemoprophylaxis even when there is a downward leprosy trend. The ranking of interventions by their predicted additional reduction relative to the baseline control is the same for simulations at 100% and 80% coverage of individuals (S2 Fig). As expected, a lower coverage level reduces the impact of the interventions. The following comparisons of interventions are at 80% coverage because it is more realistic.

Chemoprophylaxis in household contacts only leads to a slower initial reduction in cases than intervention scenarios with mass chemoprophylaxis only. The predicted number of cases under the household only strategy continues to decrease relative to the baseline control program over most of the prediction period whilst mass only strategies slow down or plateau. One round of mass chemoprophylaxis is the most ineffective intervention scenario simulated because the reduction in cases plateaus from around the year 2020. Three rounds of mass chemoprophylaxis is predicted to lead to a much greater reduction although the benefit of this also plateaus relative to strategies that include household contact chemoprophylaxis.

When interventions are compared ten years after the introduction of interventions, the ranking of interventions by increasing additional reductions relative to the baseline control program is; 1) one round of mass chemoprophylaxis; 2) household contact chemoprophylaxis only; 3) three rounds of mass chemoprophylaxis; 4) household contact chemoprophylaxis with one round of mass; 5) three rounds of mass chemoprophylaxis with household contact.

The combined interventions are more effective at preventing cases when compared ten years after introduction because of the fast initial reduction with mass chemoprophylaxis, and the ongoing nature of household contact chemoprophylaxis. Household contact chemoprophylaxis and one round of mass is predicted to lead to an additional reduction of 77.8% compared to the baseline control program, and this increases to 88.3% when three rounds are implemented.

**Fig 3:**
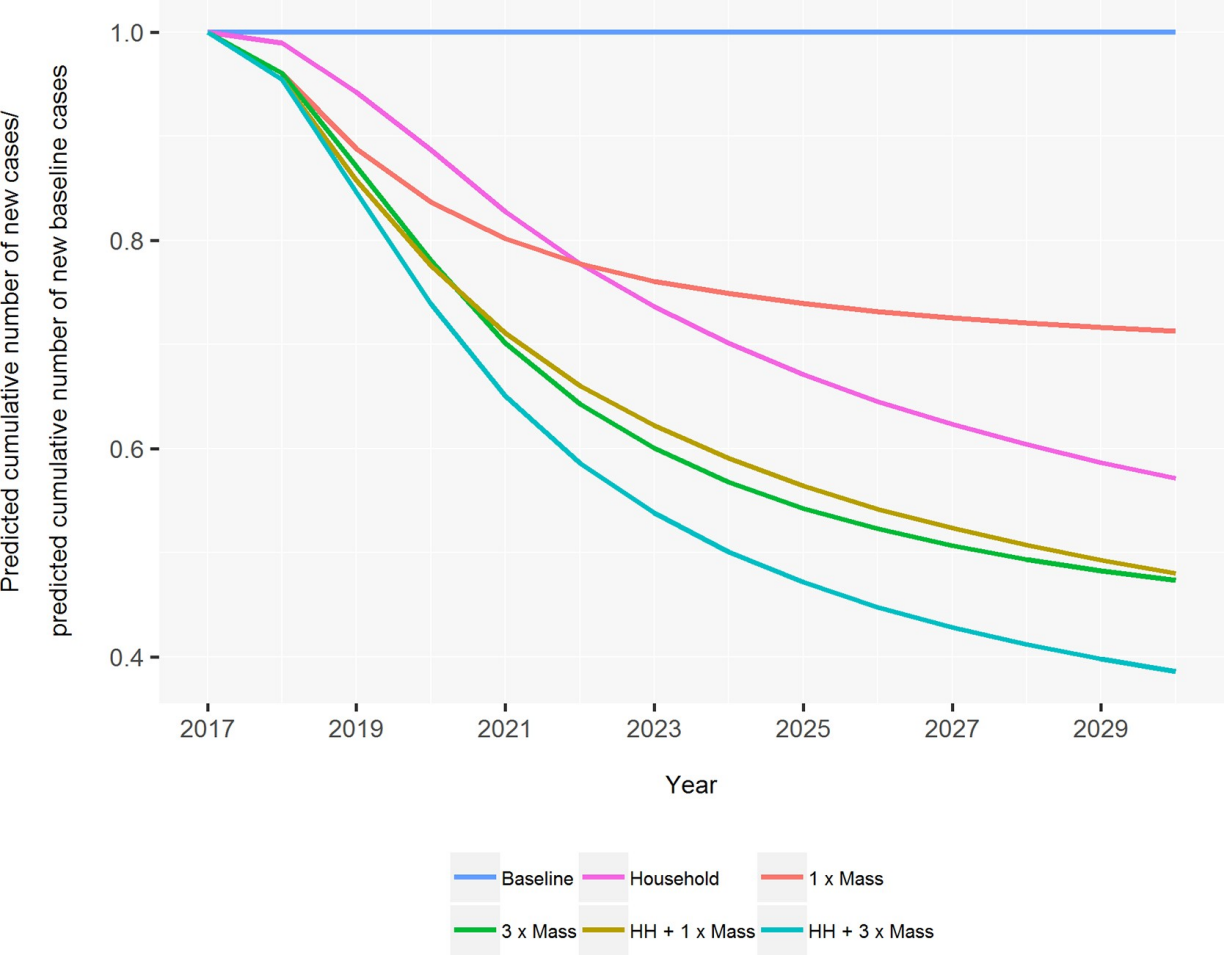
The predicted impact of six chemoprophylaxis scenarios on future cumulative cases in South Tarawa from 2017 to 2030. This figure shows the future cumulative number of cases for each chemoprophylaxis scenario relative to predictions under the baseline control program only. Results are an average of 1000 runs of the model.

Comparisons of interventions by the cumulative reduction in cases for each intervention compared to the baseline control program demonstrate that there is little difference between an intensive three rounds of mass chemoprophylaxis (49.3%) and one round with household contact (47.6%) (Fig 3). Household contact chemoprophylaxis alone is predicted to result in a reduction of 37.7%. The combined strategy of three rounds with household contact is predicted to lead to a 57.1% reduction in cumulative cases, representing the avoidance of a significant number of cases compared to the baseline control program and other chemoprophylaxis strategies.

Although at 10 years the predicted number of cases with three rounds of mass implemented every second year leads to the same annual number of cases as when implemented in consecutive years, more cumulative cases are predicted to be avoided under the latter (S3 Fig).

## Discussion

In this study, it was possible to closely replicate the leprosy trend from 1989 to 2016 using SIMCOLEP. The number of future new cases in South Tarawa are predicted to decrease substantially under the baseline control program alone, however introducing household contact chemoprophylaxis, mass chemoprophylaxis or a combination of these interventions, are predicted to lead to even more rapid reductions in new cases. The predicted reduction under the baseline control program alone highlights the benefits of the current situation of high levels of awareness of leprosy and its symptoms and therefore the importance of early case detection and treatment with the MDT recommended by WHO [20]. This high level of awareness, case detection and treatment is a prerequisite for all intervention scenarios modelled, and future cases would undoubtedly be greater if this were not to be sustained.

Mass chemoprophylaxis targets the whole population and therefore is predicted to lead to a faster reduction in cases in the first few years after implementation than household contact SDR chemoprophylaxis. Targeting the whole population has the advantage of reducing cases among more distant social and neighbourhood contacts, which make up the greater proportion of new cases when leprosy prevalence is high [8]. This modelling also demonstrated that it was beneficial to implement a more intensive approach with more than one round of mass SDR in consecutive years rather than every second year. Each additional round of rifampicin benefitted those who had been infected since the previous round and therefore were conferred no protection from the previous dose. Implementation in consecutive years prevents greater transmission in the intervening time period.

Despite household contact chemoprophylaxis leading to a slower decline, cases were predicted to continue to decline under this scenario whilst under mass strategies they plateau or even slightly increase. The benefit of household contact chemoprophylaxis is later in the prediction period when a greater proportion of new cases are in the same household as another case.

A combined strategy is likely to be needed for elimination, to rapidly reduce transmission in the general population through intensive mass chemoprophylaxis thus reducing future cumulative cases, whilst providing a sustained and timely response to household contacts who are at highest risk.

Predictions for the effectiveness of SDR chemoprophylaxis in household contacts differed in countries where SIMCOLEP modelling has previously been undertaken. Fischer et al. predicted it would lead to a 25% lower NCDR than the baseline control program at 25 years after its introduction in northwest Bangladesh [11]. De Matos et al. predicted it would lead to a 40% reduction over the baseline control program alone after 35 years in Pará State, Brazil [12]. These compare with 77.4% after 12 years predicted for South Tarawa in this study. The most likely contributing factor to these differences is the household size distributions of these regions. The average household size in Kiribati is 7.2 members compared with 4.6 members [21] and 4.1 members [22] in northwest Bangladesh and Pará State, respectively.

There are a number of limitations related to both the model and the dynamics of the spread of leprosy in Kiribati. The validity of this model relies on the accuracy of the reported number of cases in South Tarawa. The peaks in cases can be mostly explained by changes in active or passive case finding which have been implemented in the past, but not sustained. The first of these included a national screening programme in 1997 which covered the whole of Kiribati, but later case finding activities have been more limited [16]. This has contributed to uncertainty in the calibrated parameter values. For example, the passive detection delay in 1985 was very long so that it is possible that the model’s structure prevented the calibration of a shorter delay. However plausible factors such as stigma, knowledge of health care professionals and accessibility to the health care system could contribute to this delay [23].

The model assumed that only MB leprosy was infectious. Epidemiological studies suggest that PB cases could also be infectious, albeit to a lesser extent [24]. Even if PB is much less infectious than MB disease, PB cases could be a significant contributor to transmission in South Tarawa as two-thirds of cases are this form. Therefore, transmission and the number of cases in the future may be underestimated in this model but this is unlikely to impact on the ranking or trends.

An additional complication is the high level of internal migration from the outer islands to South Tarawa which could not be included in the population dynamics of the model. It was therefore not possible to determine the impact of the introduction of more cases into South Tarawa over time. It is possible that net migration into South Tarawa would favour implementing a repeated mass chemoprophylaxis strategy to catch migrants previously exposed in other islands of Kiribati.

Finally, we do not know the true mechanisms for heterogeneity of leprosy susceptibility. In this model, susceptibility was assumed to be randomly distributed. SIMCOLEP also allows for the specification of household and/or genetic mechanisms, however a previous study demonstrated that no particular mechanism was the most likely [10]. The random mechanism was found to lead to the fastest reduction and is therefore a best case scenario.

This model has provided important insights into the complex transmission dynamics of leprosy in South Tarawa. The absolute number of future new cases is predicted to be very low because although the rate is high, the Kiribati population is small. This means that the difference between predicted impacts is only a few cases between interventions. However, the primary focus of this study was to inform policy by qualitative ranking of interventions relative to the baseline control program, or to each other, rather than the absolute impact of each intervention on case numbers.

A recent article by Lockwood et al. expressed concerns regarding SDR chemoprophylaxis, in particular in close contacts [25]. They point out the obvious concern that SDR might promote rifampicin resistance while acknowledging that the actual impact of this is not known. They also mention the ethical dilemma of identifying the disease status of leprosy patients to their contacts. Disclosure of disease status in household contacts has been found to be acceptable in Bangladesh and people from Pacific Island countries living in New Zealand [26]. This concern is addressed with the use of mass chemoprophylaxis as case identification is not necessary. Lockwood et al. also cite the issues of focusing on household contacts when the COLEP study found SDR to be more effective in those with the lower baseline risk. This modelling study addressed this by comparing household contact and whole population approaches, however an underlying assumption of all interventions was that SDR cures 50% of those in the subclinical phase as it was not possible to specify different protective effects by baseline risk.

The findings of this study suggest that implementation of SDR chemoprophylaxis to household contacts of new cases, together with at least one round, and preferably more, of mass SDR would give the most rapid reduction in new cases. Associated benefits include reducing burden of disease and limiting the social consequences implicit in a diagnosis of leprosy. Despite increasing calls for a focus of leprosy control of high risk contacts [9], some populations may still benefit from a whole population approach. The findings of this study could also inform leprosy control policy in similar small but densely populated countries or regions.

## Supporting information

S1 Table Demographic and epidemiological data to quantify model. (DOCX)

S2 Table Parameters describing household movement in South Tarawa. (DOCX)

S1 Fig Observed and model household size distribution in South Tarawa. The observed distribution is the urban area household size distribution from the Kiribati Demographic and Health Survey 2009. The simulated distribution was obtained by fitting the model to this data. There is no significant difference between these two distributions (Χ^2^-test).
(TIFF)

S2 Fig The predicted impact of six chemoprophylaxis scenarios at 100% coverage on future cases in South Tarawa from 2017 to 2030. All interventions are relative to the baseline control program. Results are the average of 1000 runs.
(TIF)

S3 Fig The predicted impact of four chemoprophylaxis scenarios on future cumulative cases in South Tarawa from 2017 to 2030. Combined household contact and mass interventions relative to household contact chemoprophylaxis alone. Results are the average of 1000 runs.
(TIFF)

S4 Fig Confidence intervals of combined household contact and mass chemoprophylaxis interventions compared with the baseline control program at 2027. 95% confidence intervals obtained from 1000 model runs. The lines represent the average difference.
(TIF)

S5 Fig Confidence intervals of combined household contact and mass chemoprophylaxis interventions compared with chemoprophylaxis in household contacts only at 2027. 95% confidence intervals obtained from 1000 model runs. The lines represent the average difference.
(TIF)
