# Supplementary material for: Predicting the impact of household contact and mass chemoprophylaxis on future new leprosy cases in South Tarawa, Kiribati: A modelling study
Source: PLoS Negl Trop Dis. 2019 Sep 20;13(9):e0007646. doi: 10.1371/journal.pntd.0007646 (PMC6754131; doi:10.1371/journal.pntd.0007646)
Supplement: S1 Table — (DOCX) [file pntd.0007646.s001.docx]

**Table S1**. **Demographic and epidemiological data to quantify the model**

| **Data** | **Years** | **Source** |
| --- | --- | --- |
| **Demographic data** |  |  |
| Population growth | 1947–2010 | Kiribati Census 2010 Analytic Report [1] |
| Fraction married | 1973, 1985 and 2010 | UN WPP 2015^*^ [2] |
| Survival rates | 1905, 1990 and 2010 | UN WPP 2015 |
| Fertility rates | 1960, 2000, 2010 | UN WPP 2015 |
| Age distribution | 2015 | Kiribati Census 2015 [3] |
| Distribution of household size | 2009 | DHS 2009^†^ [4] |
| **Epidemiological data** |  |  |
| New case detection rate | 1989–2006, 2008–2016 | PLF Database^‡^ |
| Proportion of case by MB and PB | 2015 | PLF Database |
| BCG coverage | 1980–1985, 1987–2014 | WHO/UNICEF^§^ [5] |
| ^*^ United Nations World Population Prospects 2015, ^†^ 2009 Kiribati Demographic and Health Study. ^‡^ Pacific Leprosy Foundation database, ^§^ WHO and UNICEF Joint Monitoring Programme for immunization | | |

1. Kiribati National Statistics Office and the SPC Statistics for Development Programme. Kiribati 2010 Census of Population and Housing Volume 2: Analytical Report. Noumea, New Caledonia: Secretariat of the Pacific Community; 2012.

2. United Nations. World Marriage Data 2015. New York City: Department of Economic and Social Affairs, Population Division; 2015.

3. Kiribati National Statistics Office. 2015 Population and Housing Census Volume 1. Bairiki, Kiribati: Ministry of Finance; 2016.

4. Kiribati National Statistics Office (KNSO) and SPC. Kiribati Demographic and Health Survey. Noumea, New Caledonia: Secretariat of the Pacific Community; 2009.

5. WHO, UNICEF. Kiribati: WHO and UNICEF estimates of immunization coverage: 2015 Revision 2016 July 6.
