# Supplementary material for: Predicting the impact of household contact and mass chemoprophylaxis on future new leprosy cases in South Tarawa, Kiribati: A modelling study
Source: PLoS Negl Trop Dis. 2019 Sep 20;13(9):e0007646. doi: 10.1371/journal.pntd.0007646 (PMC6754131; doi:10.1371/journal.pntd.0007646)
Supplement: S2 Table — (DOCX) [file pntd.0007646.s002.docx]

**Table S2.** **Parameters describing household movement in South Tarawa**

| **Parameters** | **Values** | **Source** |
| --- | --- | --- |
| Fraction of males moving without marriage | 0.61 | Calibrated |
| Fraction of females moving without marriage | 0.0 | Fischer et al. 2010 [1] |
| Age of first movements of individuals | 12–22 years | Fischer et al. 2010 |
| Household size to move to | Start: 0.0 | Fischer et al. 2010 |
|  | End: 50.0 | Fischer et al. 2010 |
|  | Max: 12.8 | Calibrated |
| Rate of splitting households afters marriage (exponential) | 32.7 years | Calibrated |
| Fraction of widows/widowers moving to children | 1.0 | Fischer et al. 2010 |
| Household size above which widows/widowers move to children | 2.0 | Assumption |
| Fraction of males moving to partner after marriage | 1.0 | Assumption |
| Fraction of females moving to partner after marriage | 0.0 | Fischer et al. 2010 |
| Fraction of males creating a single household | 0.0 | Assumption |

1. Fischer EAJ, Vlas SJ, Meima A, Habbema DF, Richardus JH. Different mechanisms for heterogeneity in leprosy susceptibility can explain disease clustering within households. PLoS One. 2010;5.
