# Supplementary figures and images for: Predicting the impact of household contact and mass chemoprophylaxis on future new leprosy cases in South Tarawa, Kiribati: A modelling study
Source: PLoS Negl Trop Dis. 2019 Sep 20;13(9):e0007646. doi: 10.1371/journal.pntd.0007646 (PMC6754131; doi:10.1371/journal.pntd.0007646)

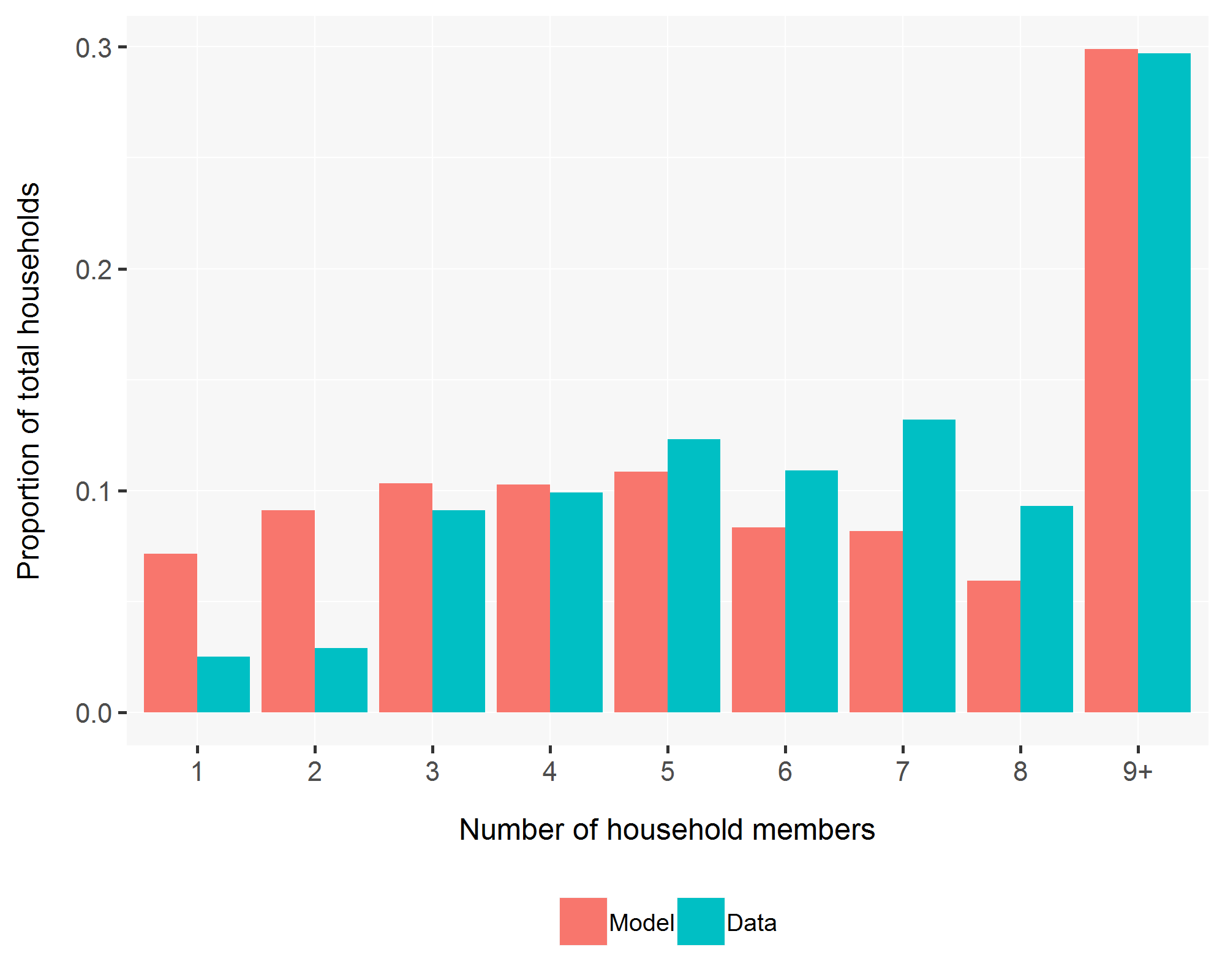

Supplement: S1 Fig — The observed distribution is the urban area household size distribution from the Kiribati Demographic and Health Survey 2009. The simulated distribution was obtained by fitting the model to this data. There is no significant difference between these two distributions (Χ2-test). (TIFF) [file pntd.0007646.s003.tiff]

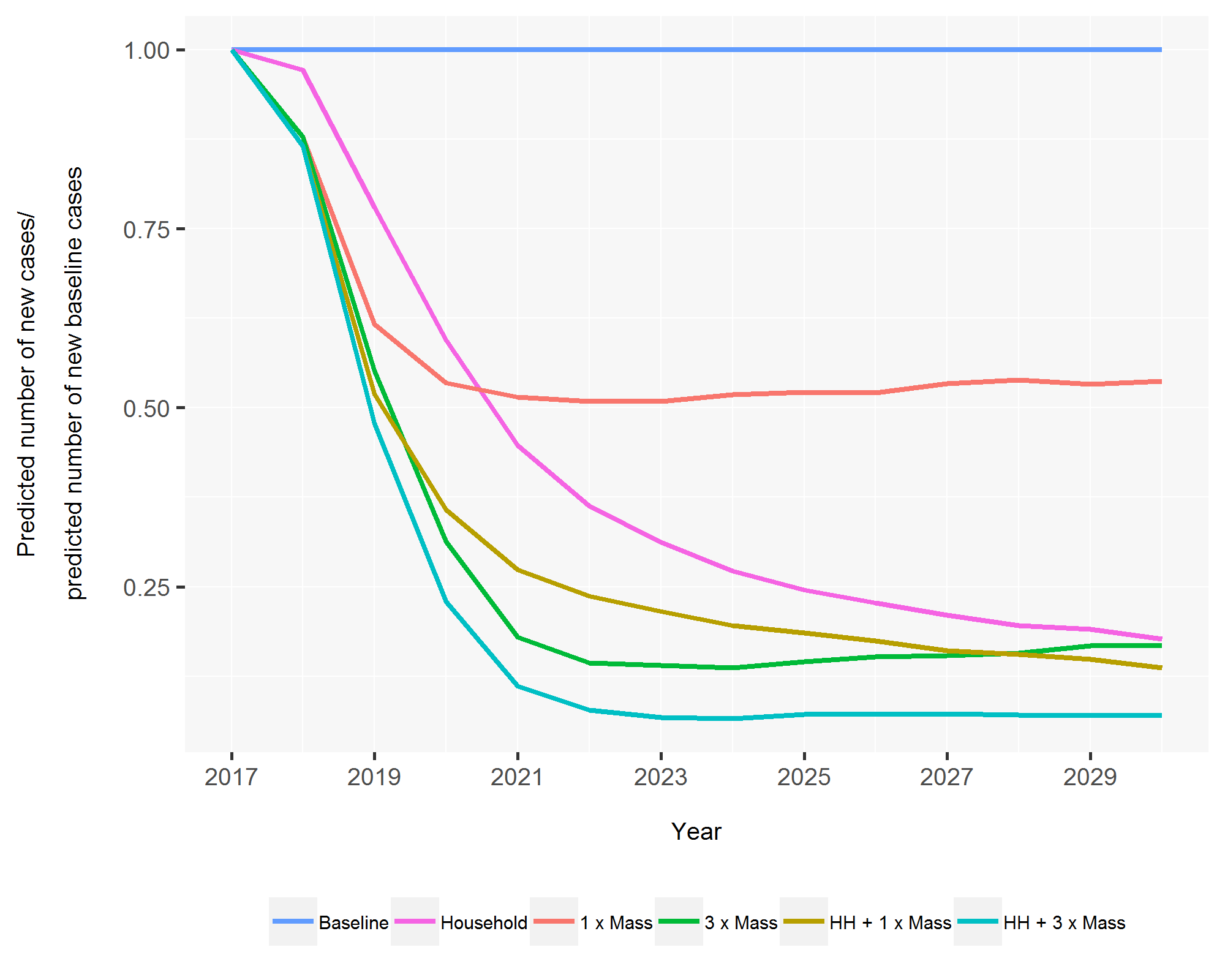

Supplement: S2 Fig — All interventions are relative to the baseline control program. Results are the average of 1000 runs. (TIF) [file pntd.0007646.s004.tif]

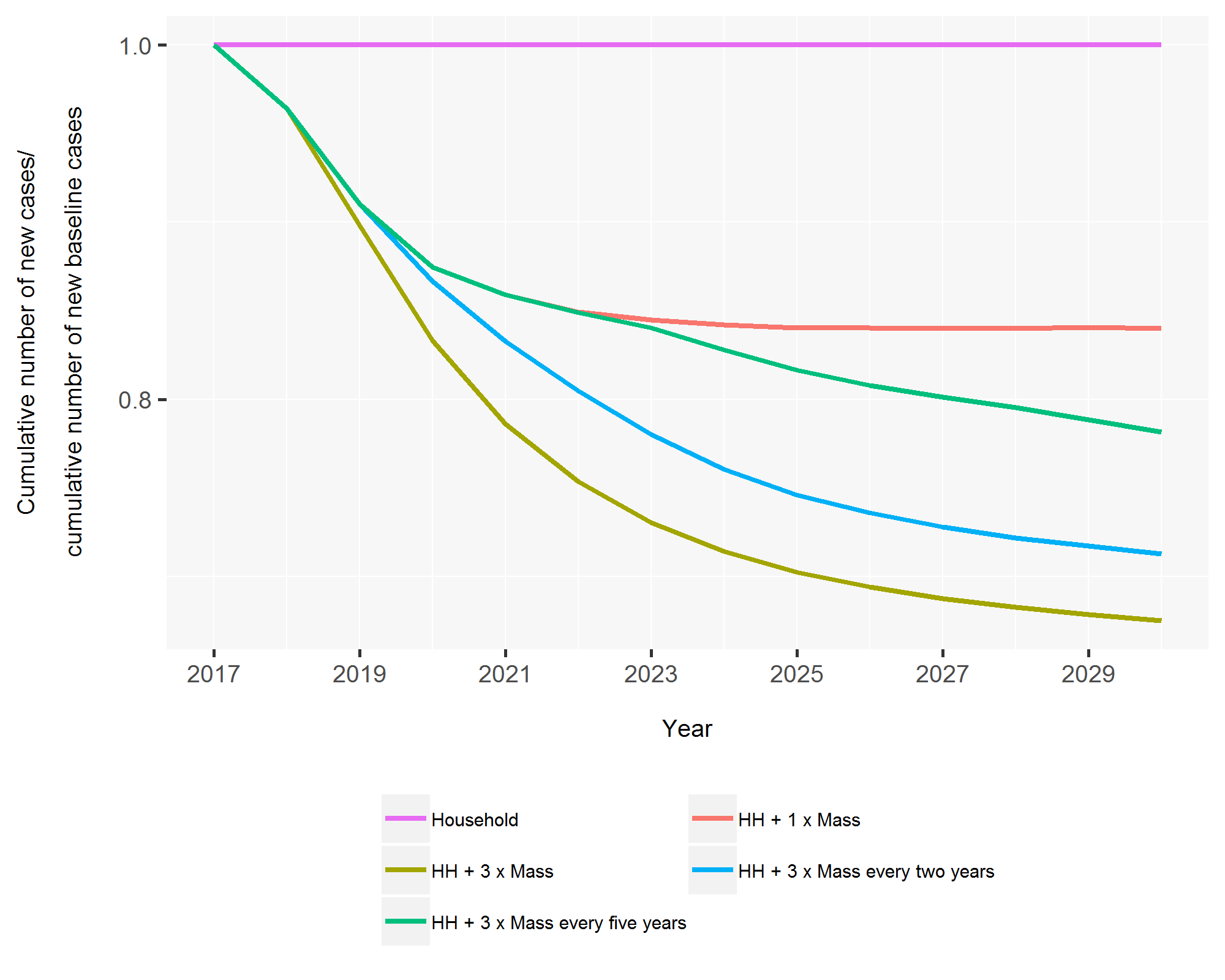

Supplement: S3 Fig — Combined household contact and mass interventions relative to household contact chemoprophylaxis alone. Results are the average of 1000 runs. (TIFF) [file pntd.0007646.s005.tiff]

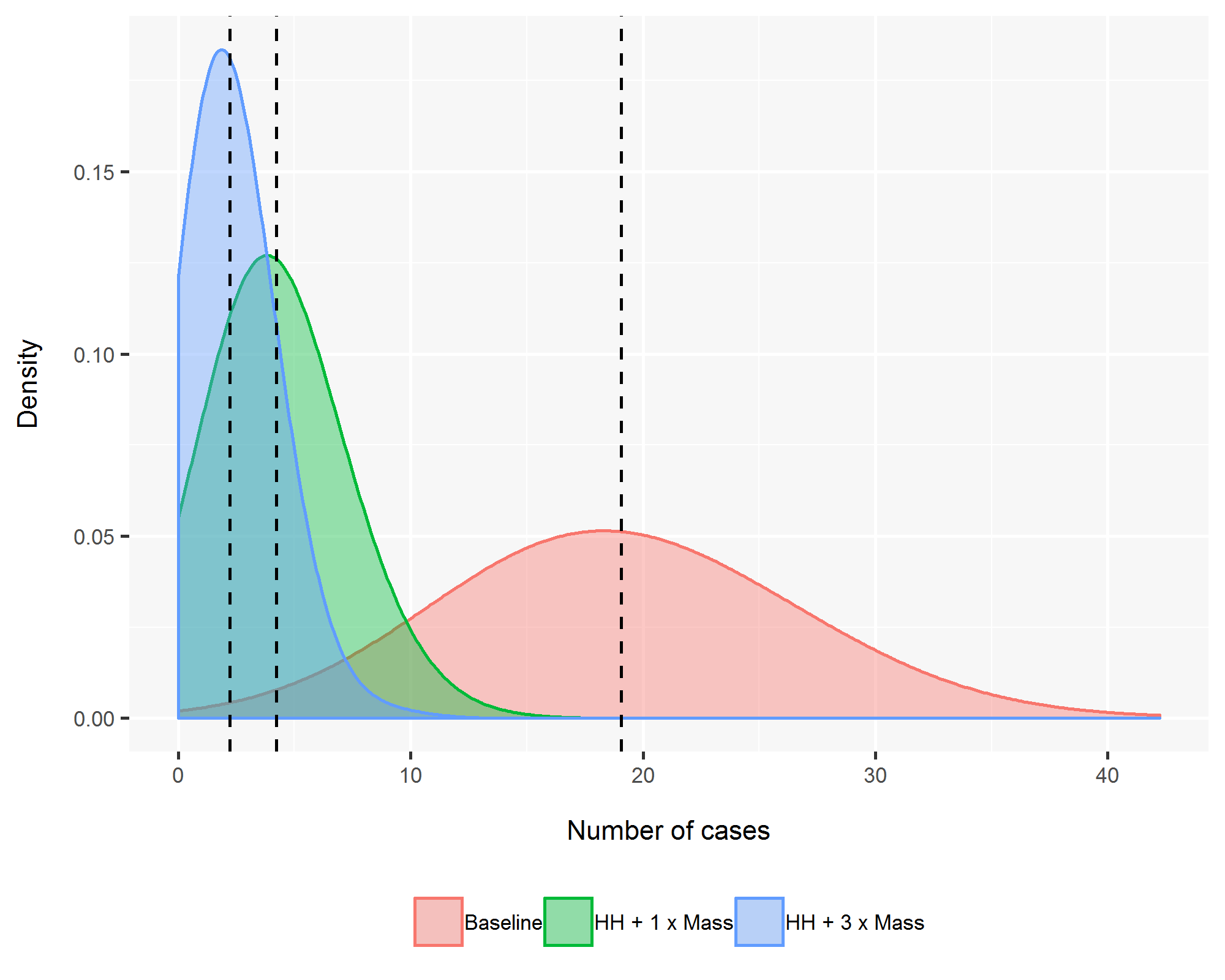

Supplement: S4 Fig — 95% confidence intervals obtained from 1000 model runs. The lines represent the average difference. (TIF) [file pntd.0007646.s006.tif]

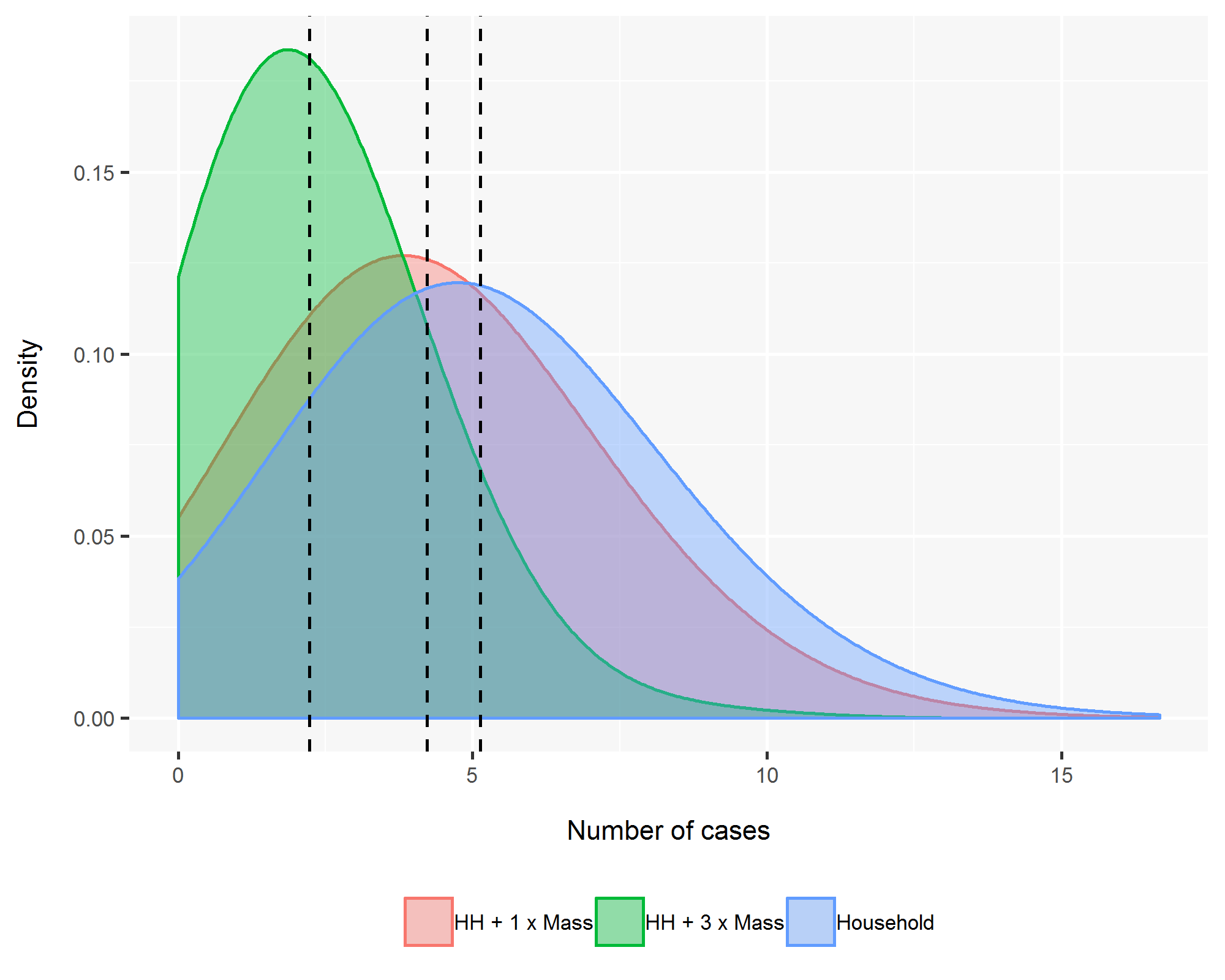

Supplement: S5 Fig — 95% confidence intervals obtained from 1000 model runs. The lines represent the average difference. (TIF) [file pntd.0007646.s007.tif]
